# Evaluation of Canine Erythrocyte Surface Antigens and Morphological Alterations Induced by Trypsin Treatment

**DOI:** 10.3390/ani15040491

**Published:** 2025-02-10

**Authors:** Yun-Joo Geum, Hyun-Jung Han

**Affiliations:** Department of Veterinary Emergency and Critical Care Medicine, College of Veterinary Medicine, Konkuk University, Seoul 05029, Republic of Korea; rmadw091@konkuk.ac.kr

**Keywords:** enzymatic conversion, trypsin, universal blood, dog erythrocyte antigen (DEA), Dal, acute hemolytic transfusion reaction

## Abstract

Dogs have multiple blood type antigens, among which DEA 1, DEA 4, and Dal can induce severe acute hemolytic transfusion reactions. Various antigen modulation techniques have been developed to reduce immunogenicity and transfusion reactions. Recently, trypsin has been suggested as a potential tool for modulating the antigenicity of DEA 1 in veterinary medicine. Following this rationale, this study aims to evaluate the effects of trypsin on the antigenicity of these three antigens. A 50% RBC suspension treated with 1 mg/mL trypsin was incubated at 37 °C for 120 min. The antigenicity of DEA 1, DEA 4, and Dal was assessed using blood typing assays before and after trypsin treatment. As a result, trypsin did not reduce the antigenicity of DEA 1 and DEA 4; instead, trypsin significantly increased their antigenicity (*p* = 0.008) and promoted agglutination, whereas Dal exhibited a significant reduction in antigenicity (*p* = 0.008). Quantitative morphological parameters obtained from an automated hematology analyzer revealed no significant differences between trypsin-treated and negative control groups. However, morphological scoring under an optical microscope showed significantly fewer echinocytes in the trypsin-treated group (*p* = 0.008). Consequently, broad-spectrum proteases like trypsin are unsuitable for universal blood production due to their variable effects on erythrocyte surface antigens.

## Simple Summary

Blood transfusions are essential for treating severe anemia and critically ill patients. However, limitations such as an insufficient supply of blood products and the risk of fatal transfusion reactions hinder their use in veterinary critical care. As a result, various techniques to eliminate or reduce the antigenicity of erythrocyte surface antigens have been investigated. In veterinary medicine, trypsin has previously been suggested to reduce the antigenicity of canine erythrocytes. However, in this report, trypsin produced variable results across three erythrocyte surface antigens. To develop universal blood products for dogs, antigen-specific enzymes are needed, rather than broad-spectrum proteases like trypsin. Therefore, a thorough understanding of the biochemical structures and interactions of these antigens should be a priority.

## 1. Introduction

Blood type is determined by antigens present on erythrocyte surfaces, with over 13 different blood types identified in dogs [1]. Several antigens are particularly significant in veterinary transfusion medicine due to their strong immunogenicity [1]. The most severe transfusion reaction, acute hemolytic transfusion reaction (AHTR), can occur with three canine erythrocyte antigens: dog erythrocyte antigen (DEA) 1, DEA 4, and Dal [2]. AHTR occurs when antibodies generated against erythrocyte antigens lead to type 2 hypersensitivity, resulting in hemolysis within minutes to hours after the transfusion [3]. Therefore, reducing alloimmunization against erythrocyte surface antigens is crucial to prevent fatal transfusion reactions [4].

In human medicine, various antigen modulation strategies have been developed to prevent transfusion reactions by modifying antigens. These strategies include enzymatic conversion of antigens, non-specific erythrocyte coating with polyethylene glycol (PEG) derivatives, and the in vitro production of erythrocytes from genetically modified stem cells [5]. The first approach to antigen modulation is enzymatic conversion [6]. Selective enzymes, such as *N*-acetylgalactosamine deacetylase and galactosaminidase, have successfully converted type A human blood to type O [7]. The second approach, erythrocyte coating—also known as stealth erythrocytes—reduces antigen expression by treating erythrocytes with PEG derivatives [8]. Various protocols for PEG treatment and different types of PEG derivatives have been actively studied to enhance the in vivo adaptation of stealth erythrocytes [5]. The final approach involves stem cell manipulation [9]. Genetically modified stem cells, designed to lack specific erythrocyte surface antigens, are differentiated into erythrocytes with reduced immunogenicity [5].

However, compared to human medicine, research in veterinary medicine remains limited due to the relatively scarce knowledge of erythrocyte surface antigens. Among the various antigen modulation strategies, only erythrocyte coating has been explored, demonstrating feasibility in creating universal blood for bovine [10] and feline erythrocytes [11].

Recently, a study suggested trypsin as a potential enzyme for antigen conversion, implicating its ability to reduce the immunogenicity of the DEA 1 antigen [12]. Therefore, this study aims to validate the potential of trypsin in modulating DEA 1 and further investigate alterations in immune responses following trypsin treatment of DEA 4 and Dal, which are also known to induce AHTR.

## 2. Materials and Methods

### 2.1. Canine Blood Sampling and Preparation

Blood samples were collected from eight healthy dogs that met the canine blood donor screening criteria [13]. The dogs were accompanied by their owners at the KU I’M DOgNOR Blood Donation Center (Konkuk University, Seoul, Republic of Korea). Following pre-donation laboratory examinations, leftover blood was used for this study. Blood samples were placed into K2-EDTA tubes (BD Vacutainer^®^ tubes, Becton Dickinson and Company, Franklin Lakes, NJ, USA) and stored at 4 °C until use. To prevent storage lesions, this study was conducted within 6 h of blood collection. The Institutional Animal Care and Use Committee of Konkuk University approved all procedures (protocol number, KU24060).

Each blood sample was centrifuged at 1000× *g* for 10 min at 4 °C and washed three times with 0.9% normal saline (NS) at 1000× *g* for 3 min. During centrifugation and washing, plasma and buffy coat were removed, resulting in packed red blood cells (pRBCs).

### 2.2. Trypsin Treatment

A portion of pRBCs was diluted with NS to prepare a 5% RBC suspension. The suspended samples were then divided into two aliquots for erythrocyte morphological analysis: one designated for the trypsin-treated group and the other for the negative control group. In the trypsin-treated group, 1 mg/mL trypsin (lyophilized trypsin from porcine pancreas, Sigma-Aldrich, St. Louis, MO, USA) was added to the 5% RBC suspension, while in the negative control group, an equal volume of NS was added instead of trypsin. Prior to use, trypsin was diluted in isotonic 1× phosphate-buffered saline at room temperature for 15 min. Both the trypsin-treated group and the negative control group were then incubated at 37 °C for 120 min with gentle shaking at 30 RPM in a heat-controlled water bath.

### 2.3. Measurement of Erythrocyte Antigenicity by Blood Typing

Blood typing was conducted before and after trypsin treatment using a 50% RBC suspension. Three canine blood types—DEA 1, DEA 4, and Dal—were tested using blood typing kits, following the manufacturer’s instructions with minor modifications. For card agglutination tests, double the amount of diluent (80 μL) was added based on further research [14] to enhance the accuracy of agglutination intensity.

For DEA 1, an immunochromatographic strip test (LabTest DEA 1, Alvedia, Limonest, France) was performed, with band intensity evaluated by the author on the following scale: 0, no band; 1+, indistinct red band; 2+, distinct red band but not as deep as the control band; 3+, deep red band like the control. For DEA 4 and Dal, card agglutination tests (RapidVet-H DEA 4 Agglutination Card Test and RapidVet-H Dal Agglutination Card Test, DMS, Flemington, NJ, USA) were conducted, with the author assessing the agglutination intensity on the following scale: 0, no agglutination; 1+, mild small agglutinates; 2+, many small agglutinates; 3+, large agglutinates; 4+, large agglutinates aggregated with each other.

### 2.4. Morphological Analysis of Erythrocytes

Morphological analysis was conducted using two methods: quantitative morphological parameters and morphological scoring of blood smear slides. Both analyses used a 50% RBC suspension. For the quantitative morphological parameters, an automated hematology analyzer, ProCyte Dx^®^ (IDEXX Laboratories, Westbrook, MA, USA), which utilizes laser flow cytometry techniques, was used. Hematocrit (HCT), hemoglobin (Hg), mean corpuscular volume (MCV), mean corpuscular hemoglobin concentration (MCHC), and red cell distribution width (RDW) were determined by ProCyte Dx^®^.

For morphological examination, blood smear slides were prepared, fixed in 100% methanol, and stained with Diff-Quik. The examination was conducted for the pre-trypsin, trypsin-treated, and negative control groups. The morphology of 500 erythrocytes was assessed using an optical microscope (CX43, Olympus, Tokyo, Japan) with an oil immersion lens at ×100 magnification. This evaluation was based on a defined morphological scoring system [15,16]. In this scoring system, a discocyte has a score of +3, an echinocyte 1 and 2 (irregular or flat erythrocytes with several spicules) has a score of +2, an echinocyte 3 and spheroechinocyte (spherical erythrocyte with several spicules) has a score of +1, and a spherocyte (spherical erythrocyte) has a score of 0. The denominated poikilocytes in the scoring system, including stomatocytes, schizocytes, and ghost cells, were also assigned a score of 0.

Additionally, autoagglutination was assessed [17] in the trypsin-treated group by mixing samples with autologous plasma and examining under the optical microscope.

### 2.5. Statistical Analysis

Statistical analysis was performed using the scipy.stats library in Python 3.12. Measured data are presented as mean ± standard deviation. Data comparisons were conducted using Wilcoxon signed rank tests, with a *p*-value < 0.05 considered statistically significant.

## 3. Results

Blood samples were collected from dogs of the following breeds: Labrador Retriever (*n* = 4), Golden Retriever (*n* = 2), Samoyed (*n* = 1), and mixed breed (*n* = 1). Four dogs were male, and four were female. Three male dogs were castrated and three female dogs were spayed. The median body weight was 32.21 kg (range, 24.5–38.7 kg), and the median age at blood donation was 3.3 years (range, 2–6 years).

**Figure 1:**
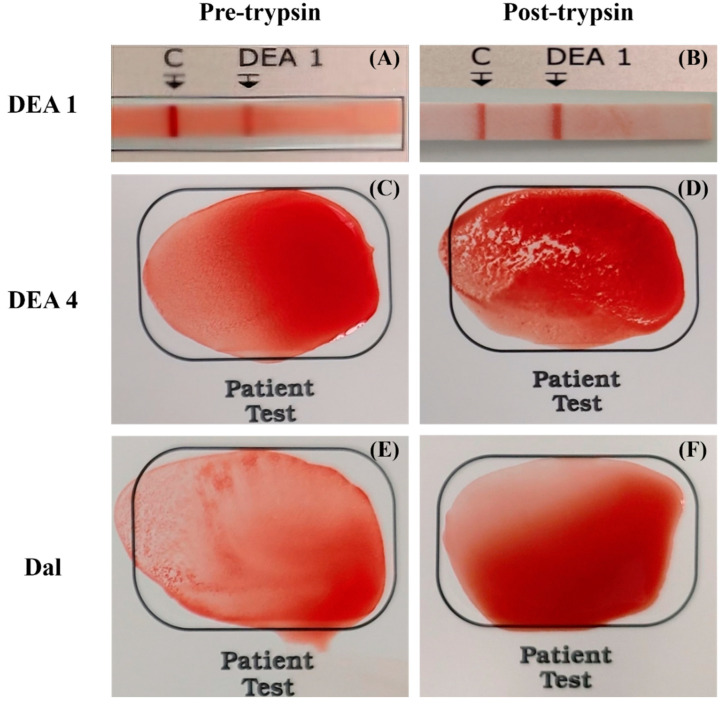
Erythrocyte antigenicity intensity of three erythrocyte surface antigens before and after trypsin treatment by using blood typing kits. (**A**) DEA 1 immunochromatographic strip test result before trypsin treatment. The test band intensity is distinct but not as deep as the control’s, and thus it is assigned a 1+ scale. (**B**) DEA 1 immunochromatographic strip test result after trypsin treatment from the same donor. The test band intensity is as deep as the control’s, indicating a +3 scale, showing increased agglutination intensity. (**C**) DEA 4 card agglutination test result before trypsin treatment. Mild small agglutinates are visible on the test card, indicating a +2 scale. (**D**) DEA 4 card agglutination test result after trypsin treatment from the same donor. Large agglutinates aggregate each other, indicating a +4 scale. (**E**) Dal card agglutination test result before trypsin treatment. Large agglutinates indicate a +3 scale. (**F**) Dal card agglutination test result after trypsin treatment from the same donor. No agglutination was observed.

Erythrocyte antigenicity intensity before and after trypsin treatment was visually assessed on a scale from 0 to 3+ for DEA 1 immunochromatographic strip tests and 0 to 4+ for DEA 4 and Dal card agglutination tests (Figure 1). Each intensity was statistically analyzed to compare before and after trypsin treatment, revealing significant differences in all three blood types—DEA 1, DEA 4, and Dal (Table 1). DEA 1 showed an intensity of 1.9 ± 0.6 before trypsin treatment, which increased to 3.0 ± 0 afterward (*p* = 0.008). DEA 4 displayed an upward trend similar to that of DEA 1; before the trypsin treatment, DEA 4 showed a similar upward trend, increasing from 2.6 ± 1.0 to 3.9 ± 0.3 (*p* = 0.008). Conversely, Dal’s intensity decreased from 2.9 ± 1.0 to 0.8 ± 0.4 (*p* = 0.008).

To evaluate the effect of trypsin on erythrocyte membrane deformity, morphological analysis results were statistically compared between trypsin-treated and negative control groups (Table 2). All quantitative morphological parameters measured by ProCyte Dx^®^ revealed no significant differences; HCT (*p* = 0.38), Hg (*p* = 0.38), MCV (*p* = 0.84), MCHC (*p* = 0.84), and RDW (*p* = 0.24). HCT and Hg were below the reference range for canine erythrocyte, while MCV, MCHC, and RDW were within the reference range.

**Figure 2:**
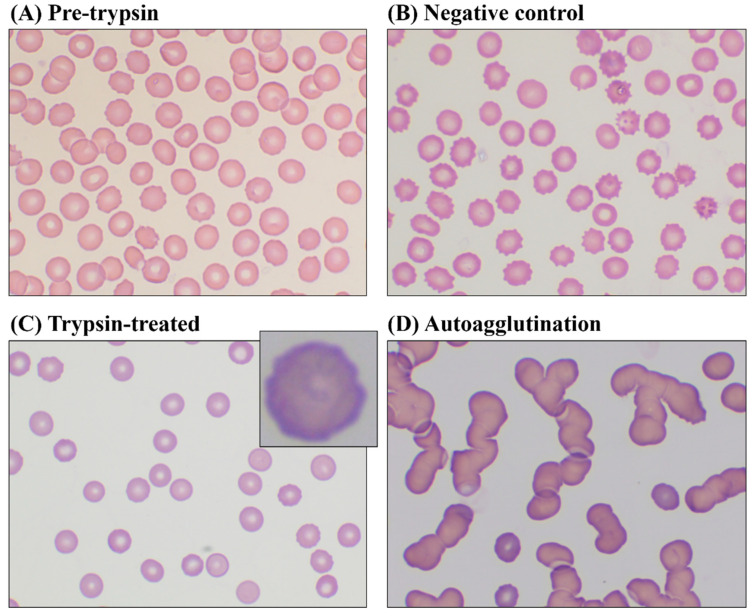
Gross examination of blood smear slides under the optical microscope before and after trypsin treatment (Diff-Quik stain, oil immersion lens at ×100 magnification). (**A**) Before trypsin treatment, blood smear slides were examined. The majority of the cells were normal biconcave discocytes. (**B**) Negative control blood samples, incubated at 37 °C for 120 min without trypsin, were examined. Echinocytes were more frequently observed compared to trypsin-treated blood samples. (**C**) Blood smear slides of trypsin-treated samples were examined. Fewer echinocytes were observed, but the erythrocyte outer membrane appeared irregular, possibly indicating a ruffled membrane. The erythrocyte in the independent box at the top right was magnified to clearly show the irregular outer membrane. (**D**) Blood smear slides of autoagglutination were examined. Two of the trypsin-treated blood samples showed autoagglutination when mixed with autologous plasma.

Morphological examination of blood smear slides under optical microscope revealed differences among pre-trypsin, trypsin-treated and negative control groups (Figure 2). The pre-trypsin group predominantly showed discocytes. The negative control group exhibited more spheroechinocytes and spherocytes, while trypsin-treated erythrocytes displayed rough and irregular outer membranes. Additionally, two trypsin-treated samples showed autoagglutination when mixed with autologous plasma, whereas no agglutination was observed in the negative control samples.

The morphological score also revealed significant differences between trypsin-treated and negative control groups (*p* = 0.008), indicating that negative control erythrocytes were less discocytic than trypsin-treated erythrocytes. The pre-trypsin group exhibited the highest morphological score (1172.6 ± 171.4).

## 4. Discussion

In this study, we evaluated DEA 1, DEA 4 and Dal—major erythrocyte surface antigens implicated in AHTR—to assess the effectiveness of trypsin in antigen modulation and its potential for universal canine blood production. We also present novel morphological alterations in canine erythrocytes following trypsin treatment.

Consequently, our results in this study indicate that trypsin is ineffective in reducing immune responses to DEA 1 and DEA 4. The agglutination intensity of DEA 1 and DEA 4 increased after trypsin treatment, while the intensity of Dal decreased. These results are highly consistent with findings from human studies. In humans, the antigen–antibody affinity of most erythrocyte surface antigens becomes stronger when treated with proteases, such as trypsin, ficin, papain and neuraminidase [18]. Several effects following protease treatment were identified [18], and two of these effects are likely associated with enhanced agglutination. First, protease cleaves sialic acid from glycoproteins on the erythrocyte extracellular surface, reducing the net negative charge and electric zeta potential, which allows cells to become closer, facilitating antibody bridging across the gap [18,19]. Trypsin has already been discovered to decrease the surface charge of human erythrocytes [20], specifically affecting only the outer membrane and not the inner membrane [21]. Second, cleavage of glycoproteins protruding from the erythrocyte surface reduces steric hindrance, further promoting antigen–antibody reactions [18]. We suggest that the stronger agglutination intensity of DEA 1 and DEA 4 observed in our study is affected by trypsin-induced glycoprotein removal, which further reduces the zeta potential and steric hindrance.

In 2021, Metheenukul reported an opposing result, indicating that trypsin successfully eliminated the antigenicity of DEA 1 [12], in contrast to the findings of this study. Two potential reasons for the conflicting results are related to variations in methodology: Fourier-transform infrared (FTIR) microspectroscopy analysis and the blood typing kits. Firstly, the FTIR results revealed a clear separation between trypsin-treated and untreated erythrocytes [12]. FTIR techniques have emerged as effective biomedical tools for diagnosing several diseases by analyzing spectral patterns, which reveal the composition and structure of biological samples [22]. Blood analysis using FTIR is also being explored; however, it is ineffective for blood typing according to the ABO and Rh systems [23]. Furthermore, assessing erythrocyte deformability requires FTIR techniques combined with morphological and rheological analyses, as deformability is influenced by several factors such as aging, high total cholesterol, and oxidative stress [24]. Therefore, while FTIR analysis reveals differences, it cannot confirm that these alterations are specifically due to DEA 1 degradation as trypsin cleaves sialic acid from the erythrocyte surface.

Secondly, the use of DEA 1 blood typing kits with different mechanisms of action may explain the discrepancies. The previous study employed card agglutination tests, while this study utilized immunochromatographic strip tests. The card test operates by detecting visible hemagglutination of DEA 1 presenting erythrocytes using lyophilized monoclonal anti-DEA 1 antibodies [25]. In contrast, similar to the enzyme-linked immunosorbent assay, the immunochromatographic strip test produces a visible test line through the capillary movement of erythrocytes across a membrane coated with monoclonal anti-DEA1 antibodies [25], and it has demonstrated reliable results in numerous reports [26,27,28]. Compared to strip tests, card-based agglutination tests generally have lower sensitivity, which increases the likelihood of false-negative results due to the difficulty in detecting weak agglutination [29]. One reason for false negatives or weak reactions, the prozone phenomenon, occurs when an excessive titer of antibodies interferes with the formation of agglutination clots [30]. To avoid this phenomenon, further dilution is recommended [30]. In 2020, Eblet et al. suggested adding more diluent to improve the accuracy of card agglutination tests for DEA 4 and DEA 5, which were produced by the same manufacturer as the DEA 1 card test [14]. Therefore, the absence of agglutination after trypsin treatment in the previous report may require further dilution to confirm whether the prozone phenomenon was a contributing factor. In this study, we applied an additional amount of diluent to the card agglutination tests for DEA 4 and Dal to prevent the prozone effect.

Proteolytic enzyme treatment is widely used in human blood group serology, as it alters antigen–antibody reactions by either destroying or enhancing them [31]. Specifically, some proteases reduce agglutination reactivity to MNS and Duffy antigens [31,32], whereas reactivity to ABO and Rh antigens is enhanced following enzyme treatment [31,33]. The MNS antigens, located on glycophorins A and B (both are single transmembrane sialoglycoproteins), can be destroyed by trypsin, chymotrypsin, ficin, and papain with variable sensitivity [32]. Similarly, Duffy antigens, situated on multiple transmembrane glycoproteins, are also susceptible to these proteases, with variable sensitivity depending on the antigenic site [32]. Both MNS and Duffy antigens show sensitivity to proteases due to the cleavage of antigen-presenting sites on human erythrocyte surface glycoproteins, resulting in the loss of specific antigens [18]. Another previous veterinary study screened canine erythrocytes against antibodies from several human blood groups—ABO, Rh, Duffy, and Kell—and reported agglutination only in response to anti-D (related to the RhD antigen), anti-Fy^a^, and anti-Fy^b^ (both related to Duffy antigens), comparing the changes in agglutination intensity before and after papain treatment [34]. Unlike human erythrocytes, papain did not weaken DEA 1 antigenicity in canine erythrocytes; instead, it produced similar or even stronger agglutination [34]. On the other hand, our study showed that Dal exhibited decreased agglutination reactions after trypsin treatment. In summary, Dal antigens are likely to have some glycoprotein structures similar to those of human Duffy antigens but lack antigenic sites that can be cleaved by papain, while trypsin effectively removes them.

The negative control group without trypsin treatment served as a baseline to determine whether observed morphological alterations in erythrocytes were specifically induced by trypsin. Quantitative morphological parameters revealed no significant differences between trypsin-treated and untreated erythrocytes, but several differences were observed under the optical microscope. Due to the optimal reaction temperature of trypsin, both pRBC samples were incubated at 37 °C for 120 min. Normal human discocytes can transform into echinocytes when incubated at 37 °C for 240 min [35]; so, the low morphological score in the negative control group may have been influenced by the incubation temperature. However, in the trypsin-treated group, significantly fewer early- and late-stage echinocytes were observed. Echinocytic spicule formation is driven by an expansion of the outer leaflet of the erythrocyte membrane bilayer and bending of the cytoskeleton [36]. The conformational changes in Band 3 glycoprotein and hemoglobin are known to adjust the cytoskeleton, mainly spectrin, thereby influencing erythrocyte deformability [37]. If the cytoskeleton is too rigid to allow for spicule formation, erythrocytes develop a ruffled edge instead [36]. In this study, echinocytes were markedly reduced; instead, irregular outer membranes, possibly indicating a ruffled edge, were observed in some trypsin-treated erythrocytes. Therefore, the small number of echinocytes in trypsin-treated samples may indicate a coarse distribution of spectrin linkages. In contrast, well-developed spectrin linkages in echinocytes form spicules, ultimately increasing erythrocyte membrane stiffness [38]. Increased stiffness signifies a loss of flexibility, making echinocytes more fragile, particularly in narrow microcirculation pathways [37]. Fragile echinocytes are known to increase during storage lesions, leading to hemolysis and reduced transfusion efficacy [37]. Based on our results, although trypsin does not reduce the antigenicity of erythrocyte surface antigens, it has been shown to decrease echinocyte formation. Consequently, further research is necessary to investigate whether trypsin can prevent echinocyte formation during storage and reduce hemolysis caused by storage lesions.

Secondly, autoagglutination occurred in two of eight trypsin-treated erythrocyte samples when mixed with autologous plasma. Protease-induced desialylation exposes additional antibody-binding sites [18], possibly leading to unpredictable immunogenic responses. In vitro trypsin-induced agglutination occurs through two sequential responses: initial incomplete antibody sensitization, followed by exposure of Thomsen-cryptantigen (T-cryptantigen) [39]. Other proteases such as papain [18] and neuraminidase [40] can also expose T-cryptantigen similarly. T-cryptantigen agglutinates with natural antibodies in almost all normal sera, causing polyagglutination, a phenomenon known as T-activation [41]. The etiology of hemolysis is thought to be related to desialylation and T-activation [41]; thus, trypsin-induced polyagglutination may be considered a potential cause of hemolysis.

Considering the findings of this study, trypsin is insufficient for enzymatic conversion. While various analyses of erythrocyte surface antigens have been conducted in humans over the past century, research in veterinary medicine remains highly limited. This lack of data complicates the selection of specific enzymes for modulating antigens. Although proteases such as ficin and neuraminidase may hold potential for modulating antigenicity, the primary focus should be on gaining a deeper understanding of the antigenic properties of the three canine erythrocyte surface antigens. Given the complexity of these antigens, it is essential to first establish a comprehensive biochemical and genetic understanding of canine erythrocyte surface antigens before selecting any enzymes. Once this foundational knowledge is established, a specific enzyme can be chosen to selectively modulate the antigens, further contributing to the production of canine universal blood.

The limitations of this study include the lack of biochemical information on canine erythrocyte antigens, which led to the use of trypsin—a non-specific enzyme that is unsuitable for reducing immunogenicity. Therefore, comprehensive investigations of canine erythrocyte surface antigens are necessary to facilitate the development of universal blood. Another limitation is that the level of glycoprotein cleavage and electrohydrodynamic effects following trypsin treatment were not analyzed. Although the primary goal of this study was to assess the morphological changes induced by trypsin, these additional analyses could provide supportive evidence and help explain the underlying mechanisms of morphological alteration. Lastly, all dogs showed the same positive result for the three blood groups (DEA 1, DEA 4, Dal), with only differences in antigenicity intensity observed. Further studies should include various blood group combinations to compare the effects of trypsin on blood group antigens.

## 5. Conclusions

Based on the results of this study, trypsin does not reduce the antigenicity of DEA 1 and DEA 4. Instead, trypsin enhances their antigenicity and promotes agglutination. Conversely, the antigenicity of Dal decreases following trypsin treatment. Therefore, broad-spectrum proteases like trypsin react differently to various erythrocyte antigens, rendering them unsuitable for the production of universal blood. To achieve universal canine blood, antigen modulation strategies focusing on antigen-specific approaches are essential. A thorough understanding of canine erythrocyte surface antigens must be quickly established in veterinary medicine.

## Figures and Tables

**Table 1:** Comparison between erythrocyte antigenicity intensity of three blood types before and after trypsin treatment.

Technique	Blood Type	Pre-Trypsin	Post-Trypsin	*p*-Value
Immunochromatographic strip	DEA 1	1.9 ± 0.6	3.0 ± 0	0.008 *
Card agglutination	DEA 4	2.6 ± 1.0	3.9 ± 0.3	0.008 *
	Dal	2.9 ± 1.0	0.8 ± 0.4	0.008 *

DEA, dog erythrocyte antigen. * *p*-value < 0.05 indicates significance.

**Table 2:** Comparison between morphological analysis of trypsin-treated and negative control group.

Variable	Reference Range	Trypsin-Treated	Negative Control	*p*-Value
**Quantitative morphological parameters**				
HCT (%)	37.3–61.7	34.13 ± 7.05	30.15 ± 6.22	0.38
Hg (g/dL)	13.1–20.5	12.16 ± 2.33	10.75 ± 1.93	0.38
MCV (fL)	61.6–73.5	66.96 ± 1.70	67.01 ± 1.14	0.84
MCHC (g/dL)	32.0–37.9	35.74 ± 0.97	35.83 ± 1.07	0.84
RDW (%)	13.6–21.7	15.26 ± 2.31	15.14 ± 2.05	0.24
**Gross examination of smear slides**				
Morphological score	0–1500	945.6 ± 160.1	639.4 ± 153.2	0.008 *

HCT, hematocrit; Hg, hemoglobin; MCV, mean corpuscular volume; MCHC, mean corpuscular hemoglobin concentration; RDW, red cell distribution width. * *p*-value < 0.05 indicates significance.

## Data Availability

The original contributions presented in the study are included in this article; further inquiries can be directed to the corresponding author.
